# Supplementary material for: Slt2-MAPK/RNS1 Controls Conidiation via Direct Regulation of the Central Regulatory Pathway in the Fungus Metarhizium robertsii
Source: J Fungi (Basel). 2021 Dec 28;8(1):26. doi: 10.3390/jof8010026 (PMC8779605; doi:10.3390/jof8010026)
Supplement: Supplementary file 1 [file jof-08-00026-s001.zip › jof-1524868-supplementary.pdf]

Table S1. Primers used in this study

Primer name	Sequence	Usage
Slt2-myc-5	GGATCCATGGCCGATCTCCAGGGA	Cloning the CDS of Slt2 to construct Slt2::Myc
Slt2-myc-3	GATATCTTACCTCCTAGATGCATCCA	
pGBKT7-Slt2-5	GGGAATTCATGGCCGATCTCCAGGG	Cloning the CDS of Slt2 for Yeast two-hybrid
pGBKT7-Slt2-3	GGGGATCCTTACCTCCTAGATGCAT	
BrlA-ChIP-5	AGTTGCCAGGCTCCAAGCTG	ChIP-qPCR analysis of *BrlA*
BrlA-ChIP-3	ATATGGAAGATGGGATGGGAC	
AbaA-ChIP-5	TTGGTGTGCTGGACCCGCTCA	ChIP-qPCR analysis of *AbaA*
AbaA-ChIP-3	TCAAGATCTATCTACACAGC	
5'RACE-F	AAGCTTGGCGTAATCCATCTCGAGCATGTG GTCGTAGT	5'RACE analysis o*f BrlA*
3'RACE-F	GATTACGCCAAGCTTCACACAGCCTATCCG GATCCGAG	3'RACE analysis of BrlA
EMSA-BrlA-*BM2*-F	CTCGACTCTTGACTCTCTCGACCAGACTCT TGACTCTTGACGCTCGACT	Bio-labeled/unlabeled probes for EMSA analysis
EMSA-BrlA-*BM2*-R	AGTCGAGCGTCAAGAGTCAAGAGTCTGGT CGAGAGAGTCAAGAGTCGAG	
EMSA-AbaA-*BM2*-F	TGCCCGTTGGGTTTTACGACAAGACCTTC ATTGGCCGGAGCTCTCGTCT	Bio-labeled/unlabeled probes for EMSA analysis
EMSA-AbaA-*BM2*-R	AGACGAGAGCTCCGGCCAATGAAGGTCTT GTCGTAAAACCCAACGGGCA	
RNS1-S306A-mutant-F	CAGAGGAAACGCTCCCTACATGGACACGC AGCG	Substituting the Ser-306 to alanine in RNS1 protein.
RNS1-S306A-mutant-R	GAGGCGTACCTGTGCCCC	
BrlA-RT-F-1	CGACTACCATCGAGATT	RT-PCR analysis for BrlAα
BrlA-RT-R-1	CGAAAAGAGGACGGACG	RT-PCR analysis for BrlAα and BrlAβ
BrlA-RT-F-2	CACACAGCCTATCCGGATCC	
BrlA-RT-R-2	ATCATTTTTCGATGGGCGCG	
AbaA-RT-F	CATTCTTAGCATGGCGGTGC	RT-PCR analysis of AbaA
AbaA-RT-R	TGCTACCAGCTCTCTCGAGT	
WetA-RT-F	CGAAATAGGAAAGCAGCCGC	RT-PCR analysis of WetA
WetA-RT-R	GTCATCACACTGCCCATGGA	
RNS1-RT-5	CCGACATCAAGCAGGACATG	RT-PCR analysis of RNS1
RNS1-RT-3	CTTGTTCTTGATGCCTCGGG	

Act-RT-F	TCCTGACGGTCAGGTCATC	Reference gene for qRT-PCR analysis
Act-RT-R	CACCAGACATGACGATGTTG	
Tef-RT-F	CTGGTACAAGGGTTGGGAGA	Reference gene for qRT-PCR analysis
Tef-RT-R	TACACATCCTGGAGGGGAAG	

Table S2. Relative germination inhibition of the WT strain, the mutant *ΔRns1* and the complemented strain *C-ΔRns1* under the four abiotic stresses.

Strains	UV radiation	Hyperosmotic	Oxidative stress	Congo red
**WT**	0.18±0.07[a]	0.38±0.02[a]	0.04±0.01[a]	0.33±0.01[a]
ΔRns1	0.14±0.05[a]	0.34±0.05[a]	0.07±0.03[a]	0.25±0.04[a]
**C-*ΔRns1***	0.25±0.05[a]	0.35±0.03[a]	0.06±0.05[a]	0.31±0.03[a]

Note: within each column, different letters represent significant difference ($P < 0.05$, Tukey's test One-way ANOVA).
